# Bacillus Calmette–Guérin-Induced Human Mast Cell Activation Relies on IL-33 Priming

**DOI:** 10.3390/ijms23147549

**Published:** 2022-07-07

**Authors:** Karen M. Garcia-Rodriguez, Anu Goenka, Darren D. Thomson, Rajia Bahri, Chiara Tontini, Barbora Salcman, Rogelio Hernandez-Pando, Silvia Bulfone-Paus

**Affiliations:** 1 Lydia Becker Institute of Immunology and Inflammation, School of Biological Sciences, Faculty of Biology, Medicine and Health, University of Manchester, Manchester M13 9PL, UK; karey1199@hotmail.com (K.M.G.-R.); d.d.thomson@exeter.ac.uk (D.D.T.); rajia.bahri-2@manchester.ac.uk (R.B.); chiara.tontini@postgrad.manchester.ac.uk (C.T.); barbora.salcman@manchester.ac.uk (B.S.); 2 School of Materials, Faculty of Science and Engineering, University of Manchester, Manchester M13 9PL, UK; 3 School of Cellular and Molecular Medicine, University of Bristol, Bristol BS8 1TH, UK; anu.goenka@bristol.ac.uk; 4 MRC Centre for Medical Mycology, University of Exeter, Exeter EX4 4PY, UK; 5 Experimental Pathology Section, Department of Pathology, National Institute of Medical Sciences and Nutrition “Salvador Zubirán”, Mexico City 14080, Mexico; rhdezpando@hotmail.com

**Keywords:** mast cells, BCG, IL-33, vaccine booster, *M. bovis*

## Abstract

Bacillus Calmette–Guérin (BCG) vaccine is an attenuated strain of *Mycobacterium bovis* that provides weak protection against tuberculosis (TB). Mast cells (MCs) are tissue-resident immune cells strategically that serve as the first line of defence against pathogenic threats. In this study, we investigated the response of human MCs (hMCs) to BCG. We found that naïve hMCs exposed to BCG did not secrete cytokines, degranulate, or support the uptake and intracellular growth of bacteria. Since we could show that in hMCs IL-33 promotes the transcription of host-pathogen interaction, cell adhesion and activation genes, we used IL-33 for cell priming. The treatment of hMCs with IL-33, but not IFN-**γ**, before BCG stimulation increased IL-8, MCP-1 and IL-13 secretion, and induced an enhanced expression of the mycobacteria-binding receptor CD48. These effects were comparable to those caused by the recombinant *Mycobacterium tuberculosis* (*Mtb*) 19-KDa lipoprotein. Finally, stimulation of hMCs with IL-33 incremented MC-BCG interactions. Thus, we propose that IL-33 may improve the immunogenicity of BCG vaccine by sensitising hMCs.

## 1. Introduction

Bacillus Calmette–Guérin (BCG) is currently the only approved and widely used vaccine against tuberculosis (TB), which is caused by *Mycobacterium tuberculosis* (Mtb) [1]. While BCG is effective at protecting against the most severe and disseminated forms of TB, it has limited efficacy in preventing pulmonary TB [2]. There are variable levels of evidence supporting the use of BCG across diverse pathologies [3], such as autoimmune disorders (e.g., multiple sclerosis and type 1 diabetes) [4], anaphylaxis [5], bladder cancer [6], urticaria [7], and cutaneous melanoma [8]. More recently, BCG vaccination has been associated with trained immunity and protective effects against parasitic, fungal, viral, and bacterial infections [9]. Trained immunity associated with BCG vaccination has also been proposed as protective measure against severe COVID-19 [10]. Furthermore, evidence suggests that BCG vaccination may reduce the incidence of atopic and inflammatory diseases during childhood [4]. Mtb antigens expressed at the bacterial wall, such as the 19-kDa lipoprotein, triggering TLR2-induced signaling and IL-2 release in macrophages, have been used to increase the immunogenicity of the BCG vaccine [11,12]. Similarly, the Heat Shock Protein 70 (HSP70) antigen promotes innate and adaptive immunity when used as vaccine adjuvant [13].

The cellular and molecular mechanisms underlying the immunomodulatory properties of BCG have not been fully elucidated. Upon vaccination, the first BCG-host immune encounters take place in the dermis where innate cells including dendritic cells (DC), macrophages (Mφ) [14], and mast cells (MCs) reside [1].

MCs are tissue-resident immune cells present at sites of host-environment interactions, such as the skin or lung mucosa [15]. Upon activation, MCs release a wide variety of pro- and anti-inflammatory mediators including cytokines, chemokines, proteases, and anti-microbial compounds [16]. MC activation is enhanced by cell exposure to specific cytokines, such as IL-33 [17]. IL-33 is an alarmin, that belongs to the IL-1 family cytokines, and it is produced by endothelial, epithelial cells, and fibroblasts in the skin, lung, and gastrointestinal tract [18]. Furthermore, IL-33 amplifies MC activities during infection and allergy [19].

To date, little is known about the means of BCG-MCs interactions. In studies on mice, MCs have been shown to phagocytose bacteria, degranulate, and form extracellular traps in response to BCG [20]. Furthermore, evidence obtained with rat MCs infected with Mtb indicates CD48 as the main receptor responsible for Mtb-MC membrane binding [21]. However, exactly which MC activities are induced by BCG infection remain unclear. Increased IL-17+ MCs are associated with a beneficial clinical outcome following BCG-treatment of patients with bladder cancer [6,22]. In these settings, the IL-17 released from MCs upon BCG exposure induce an IL-8-mediated neutrophil recruitment and tumor suppression [23]. In addition, since BCG polysaccharide nucleic acid (BCG-PSN) [7] and BCG extracts [5] suppress MC degranulation, they have been tested as a potential treatment for individuals with urticaria and in anaphylaxis mouse models.

Here, we investigate the effects of IL-33 priming on BCG-mediated hMC activation. Our findings demonstrate that, while naïve hMCs are not activated by BCG, the exposure to IL-33, but not to IFN-**γ**, renders them responsive to BCG stimulation. IL-33-primed hMCs release cytokines such as IL-8, MCP-1, and IL-13, as well as upregulate the expression of CD48, a receptor likely to support BCG-cell interactions. Thus, we suggest that a microenvironment rich in IL-33 is key to promote BCG-induced MC responses.

## 2. Results

### 2.1. Human Mast Cells Are Resistant to BCG-Induced Activation

**Figure 1:**
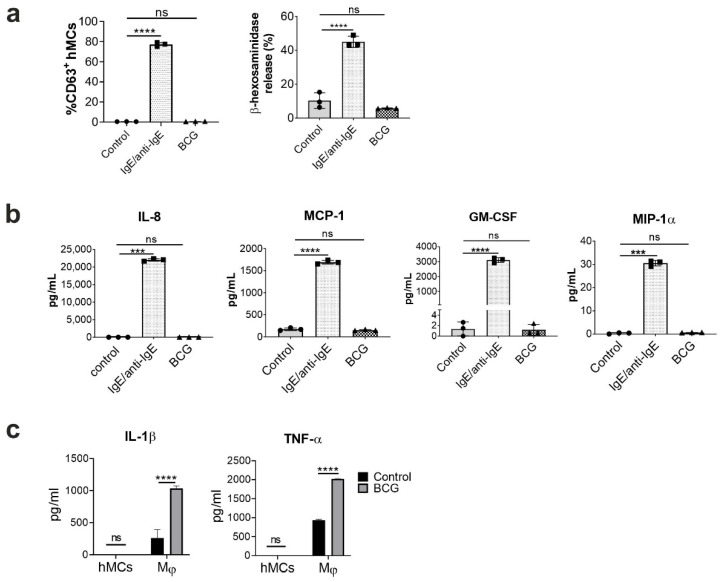
BCG does not induce MC activation. Human mast cells (hMCs, 0.5 × 10^6^ cells/mL) were stimulated with BCG (MOI 10:1) for 1 or 16h. IgE-sensitized cells activated with anti-IgE antibodies (IgE; 1 µg/mL, anti-IgE; 1 µg/mL) were used as a positive control. (**a**) After 1 h incubation, cell degranulation was measured by CD63 antibody staining (% of CD63+ cells) and β-hexosaminidase release. (**b**) Cytokine secretion was measured in supernatants collected after 16h incubation using CBA. (**c**) THP-1 cells (Mϕ) were used as positive control for BCG-induced cytokine release. Each graph is representative of 3 independent experiments. Statistical analyses were performed with one-way ANOVA analysis followed by a Tukey’s multiple comparison test (**** *p* < 0.0001, *** *p* < 0.001, ns, not significant).

Since MCs were shown to release mediators upon encounter with mycobacteria (including Mtb) [16,24], we aimed to investigate whether the BCG vaccine elicits hMC degranulation and cytokine secretion. hMCs were differentiated from circulating blood progenitors, with purity and maturity assessed by measuring CD117 expression, along with the ability to degranulate (% of CD63+ cells and β-hexosaminidase release), and secrete cytokines (IL-8, MCP-1, GM-CSF) upon FcεRI-IgE crosslinking (Supplementary Figures S1 and S2). Mature MCs were exposed to BCG at a multiplicity of infection (MOI) of 10:1 for 1 h to investigate cell degranulation, and 16h to measure cytokines and chemokines release. BCG did not significantly affect hMC degranulation, measured by the expression of CD63 (% of CD63+ cells) and by β-hexosaminidase release (% of total release), compared to untreated cells (Figure 1a). FcεRI-IgE crosslinking was used as positive control of degranulation. Similarly, secretion of IL-8, MCP-1, GM-CSF and MIP-1α (Figure 1b) was not significantly induced by BCG when compared to unstimulated controls. THP1 macrophage cells were used as a positive control for significant TNF-α and IL-1β release in their supernatants when infected by BCG (Figure 1c). Thus, BCG infection (10:1 MOI) of hMCs does not induce cell activation, measured by means of degranulation and cytokine secretion.

### 2.2. Human MCs Do Not Internalize and Promote BCG Intracellular Growth

**Figure 2:**
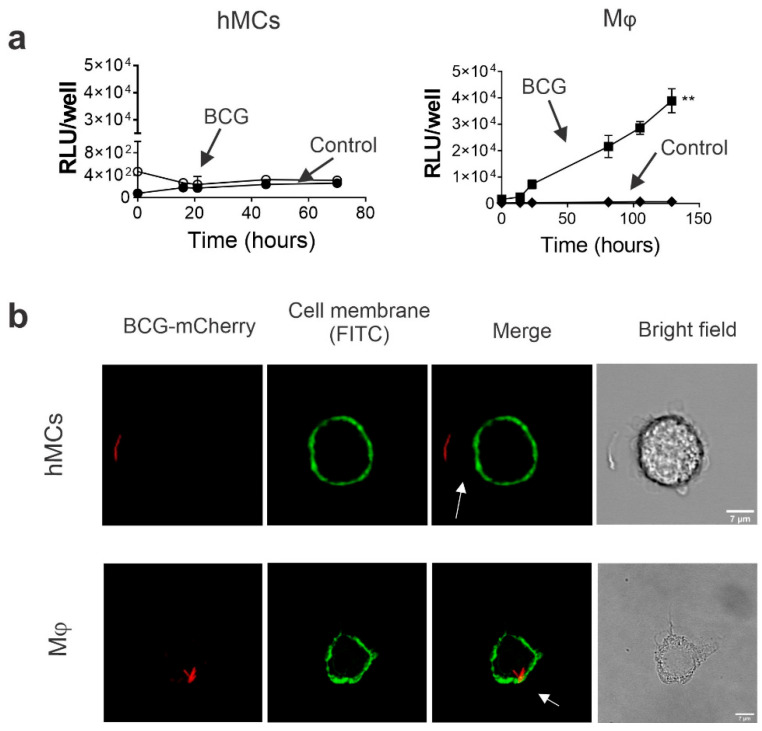
BCG does not show intracellular presence and growth in hMCs. (**a**) hMCs (left) and THP-1 cells (Mϕ) (right) (both at 0.5 × 10^6^ cells/mL) were incubated with BCG-LUX (MOI 10:1) for 2 h and washed to measure intracellular growth at different time points as increase of relative light units (RLU). Each graph is representative of 3 independent experiments. Statistical analysis was performed using the Man-Whitney test (** *p* < 0.01). (**b**) hMCs and THP-1 cells (Mϕ) (both at 0.5 × 10^6^ cells/mL) were incubated for 2 h with BCG-mCherry (MOI 10:1), fixed and stained with a FITC-fluorescent membrane dye. BCG uptake was analysed using confocal microscopy. The upper (hMCs) and lower (Mϕ) panel show representative micrographs at 40× of cells (green) and BCG (red) interaction.

To clarify the lack of a BCG-induced hMC response, we investigated the ability of hMCs to support BCG internalization and intracellular growth. To this purpose, hMCs and THP-1 macrophage cells (Mϕ) (used here as positive control) were exposed to an auto luminescent BCG strain, BCG-Lux, for 2 h at a MOI 10:1. After incubation, cells were washed to remove extracellular bacteria, and relative light units (RLUs) of bacterial luminescence were measured during incubation. BCG-Lux-infected THP-1 macrophages showed a significant increase in RLUs at all measured time points. Thus, this indicates significant intracellular BCG growth. However, no change in RLUs was detected in hMCs (Figure 2a). Furthermore, to investigate the ability of hMCs to internalize bacteria, cells were incubated with BCG mCherry (MOI 10:1) for 2 h, fixed, and analysed by confocal microscopy. Although bacteria were proximal to hMCs, they were not observed intracellularly or at the cell membrane (Figure 2b). Thus, in vitro hMCs do not exhibit BCG uptake nor support intracellular bacteria growth, unlike macrophages.

### 2.3. The Mtb 19-KDa Lipoprotein Induces CD48 Expression and Cytokines Secretion in hMCs

**Figure 3:**
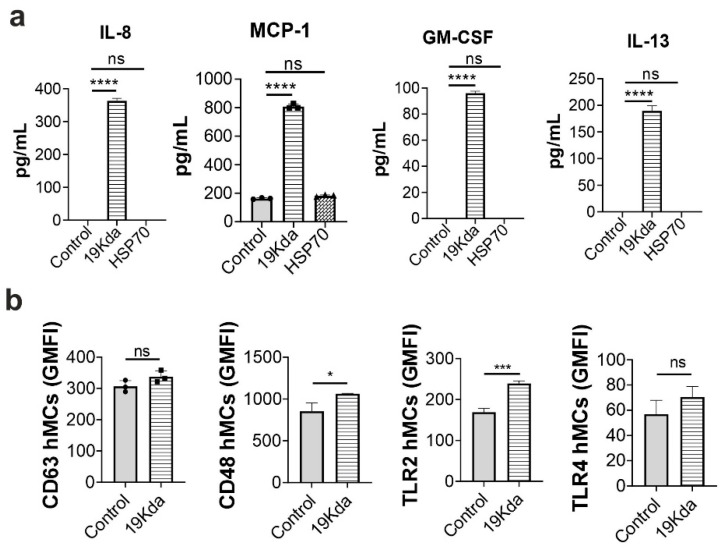
The 19-KDa lipoprotein Mtb antigen induces mediator release and increases CD48 expression in hMCs. (**a**) hMCs were stimulated with 19-KDa (10 µg/mL) or HSP70 (10 µg/mL) antigens for 16h. Supernatants were collected and the release of mediators was analysed using the CBA multiplex assay. (**b**) To investigate receptor expression and degranulation, hMCs were incubated with 19-kDa for 1 h. After incubation cells were washed, immunostained with CD63, CD48, TLR2, and TLR4 antibodies and analysed by flow cytometry. Each graph is representative of 3 independent experiments. Statistical analyses were performed with one-way ANOVA followed by a Tukey’s multiple comparison test and student t-test respectively (**** *p* < 0.0001, *** *p* < 0.001, * *p* < 0.1, ns, not significant).

Since the BCG vaccine does not activate naïve hMCs, we investigated whether virulent, highly immunogenic Mtb antigens, such as the 19-kDa lipoprotein and the 70 kDa heat shock protein (HSP70) [12,25], could stimulate hMC responses. hMCs were incubated with the 19-kDa lipoprotein and HSP70 for 16h and IL-8, MCP-1, GM-CSF, and IL-13 were measured in cell supernatants. 19-kDa-treated hMCs supernatants contained significantly increased concentrations of IL-8, MCP-1, GM-CSF, and IL-13 compared to untreated controls. However, HSP70 stimulation showed no significant effect on hMC mediators release (Figure 3a). Since 19-kDa activates hMCs by causing cytokine release, we further investigated whether 19-kDa could influence cell degranulation and bacteria-binding receptor expression. hMCs were incubated with 19-kDa for 1 h, stained with anti-CD63, anti-CD48, anti-TLR2, and anti-TLR4 fluorescent antibodies, and analysed by flow cytometry to evaluate receptor expression. As shown in Figure 3b, degranulation measured by CD63 expression was not induced by 19-kDa when compared to unstimulated controls. Furthermore, the 19-kDa lipoprotein significantly increased the expression of both CD48 and TLR2 in hMCs, with expression levels of CD48 (GMFI 1067, SD 5.57) considerably higher than TLR2 (GMFI 239, SD 6.5), and showed a trend in enhancing TLR-4 expression (although not significant). Thus, the virulent 19-kDa lipoprotein induces hMC cytokine secretion and increases the expression of the mycobacteria-binding receptor CD48, thereby suggesting that hMC unresponsiveness to BCG could be a result of the low or lacking expression of key MC activating antigens.

### 2.4. IL-33 Priming Alters hMC Responses upon BCG Exposure

**Figure 4:**
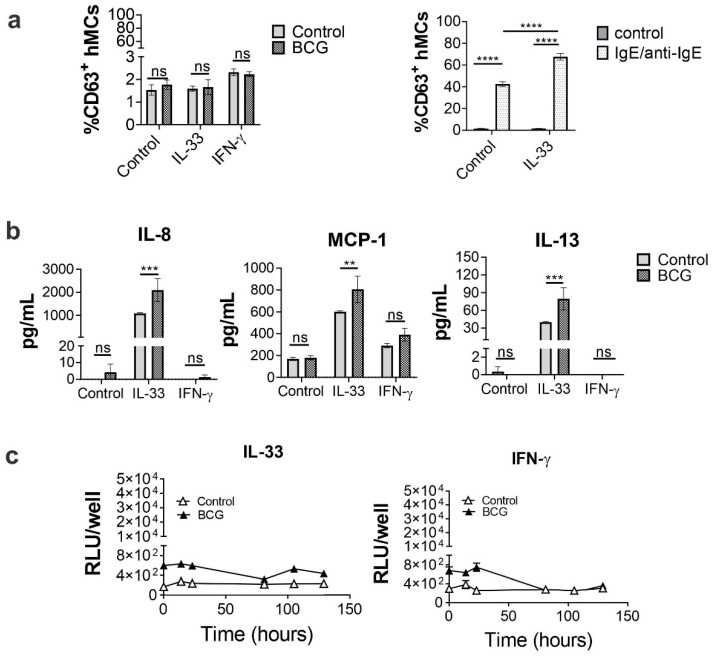
hMC cytokine secretion upon BCG infection is enhanced by IL-33. hMCs were treated with either IL-33 (50 ng/mL) or IFN-γ (50 ng/mL) for 24 h or left untreated (control). Cells were then washed and stimulated with BCG (MOI 10:1) for (**a**) 1 h to measure degranulation (% CD63+ cells) or (**b**) 16h to evaluate cytokine secretion in supernatants using CBA multiplex assay. IgE/anti-IgE stimulation (IgE; 100 ng/mL, anti-IgE; 100 ng/mL) was used as a positive control of degranulation. (**c**) IL-33 (left) and IFN-γ (right) treated hMCs were incubated with BCG-LUX (MOI 10:1) for 2 h, washed, and incubated to measure intracellular growth at different time points as an increase of relative light units (RLU). Each graph is representative of 3 independent experiments. Statistical analyses were performed with one-way ANOVA analysis followed by a Tukey’s multiple comparison test and Man-Whitney test respectively (**** *p* < 0.0001, *** *p* < 0.001, ** *p* < 0.01, ns, not significant).

IL-33 is known to potentiate hMC mediators’ secretion [26], whereas IFN-**γ** is shown to boost hMC pro-inflammatory activities upon *Staphylococcus aureus* infection [27] and LPS stimulation, as shown in Supplementary Figure S2. Because of the lack of hMC responses upon BCG exposure, we investigated whether cytokines known to activate MCs, such as IL-33 or IFN-**γ**, would promote the ability of hMCs to uptake BCG or influence BCG-mediated cell activation. As shown in Supplementary Figure S2, we initially tested IL-33 or IFN-γ priming in both LPS and FcεRI-IgE crosslinking, to evaluate cytokine responses in primed cells. This was followed by investigating cytokine secretion, degranulation, and intracellular BCG growth in hMCs incubated for 24 h in media containing either IL-33 (50 ng/mL) or IFN-**γ** (50 ng/mL), and then stimulated with BCG (MOI 10:1) for 1 or 16h to evaluate degranulation (% CD63+ cells) and cytokine secretion, respectively. IgE/anti-IgE-mediated (100 ng/mL) activation was used as positive control for degranulation in IL-33 pre-treated cells (Figure 4a). As shown in Figure 4b, IL-33 priming significantly increased hMC secretion of IL-8 (2090.6 ± 495 pg/mL), MCP-1, and IL-13, whereas cytokine levels in IFN-**γ**-primed cell supernatants were comparable to untreated controls. However, while IgE/anti-IgE degranulation was enhanced by IL-33 treatment, the latter was not sufficient to induce BCG-mediated degranulation in hMCs (Figure 4a). To investigate whether IL-33 or IFN-**γ** priming influences BCG intracellular growth, hMCs were incubated with BCG-Lux (MOI 10:1) for 2 h. After incubation, cells were washed to remove extracellular bacteria and RLUs were measured at different incubation times. As shown in Figure 4c, IL-33 and IFN-**γ** pre-treatments did not induce any increase in RLUs in hMCs; therefore, they did not promote BCG intracellular growth. Thus, hMCs treated with IL-33 respond to BCG stimulation by secreting IL-8, MCP-1 and IL-13, but do not degranulate or support intracellular bacterial growth.

### 2.5. IL-33 Enhances hMC CD48 Expression

**Figure 5:**
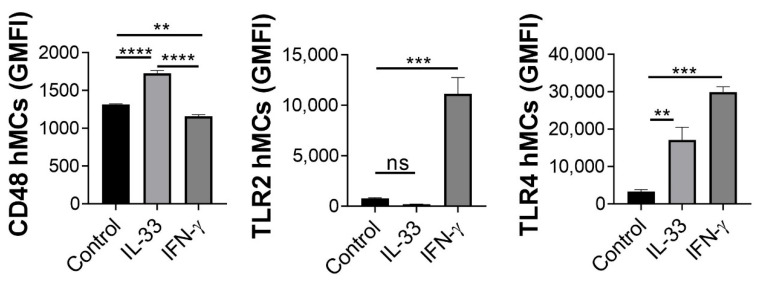
IL-33 treatment increases CD48 expression in hMCs. hMCs were incubated 24 h with IL-33 (50 ng/mL), IFN-γ (50 ng/mL) or left untreated (control), washed, stained with CD48, TLR2, and TLR4 antibodies conjugated to fluorescent fluorochromes (FITC, CD647 and PE respectively) and analysed by flow cytometry. Each graph is representative of 3 independent experiments. Statistical analysis was performed with one-way ANOVA followed by a Tukey’s multiple comparison test (**** *p* < 0.0001, *** *p* < 0.001, ** *p* < 0.01).

Since IL-33 treatment promoted cytokine release upon BCG infection, and CD48 was shown to be involved in murine MC activation by mycobacteria [21], we hypothesised that IL-33, similar to 19-kDa, could modulate bacteria-binding receptor expression. To prove this hypothesis, we incubated hMCs for 24 h with either IL-33 or IFN-**γ**, then washed and incubated with anti-CD48, anti-TLR2 and anti-TLR4 antibodies conjugated to fluorescent fluorochromes. Receptor expression (GMFI) was analysed by flow cytometry. As observed in Figure 5, CD48 expression was significantly induced by IL-33 compared to control and to IFN-**γ.** Furthermore, IFN-**γ** upregulated both TLR2 and TLR4, while IL-33 treatment showed increased expression of TLR4, but not TLR2, compared to control (Figure 5). Thus, the increase in CD48 receptor expression in hMCs correlated with an increase in mediators release upon IL-33 priming followed by BCG stimulation.

### 2.6. IL-33 Increases CD48, NOD2, ICAM-1 and MHC Class II mRNA Transcription in hMCs

To assess the effect of IL-33 on the expression of BCG-interacting and hMCs activation molecules, we performed mRNA extraction and next generation sequencing of hMCs (0.5 × 10^6^ cells/mL) treated for 24 h with 50 ng/mL IL-33 (IL-33) or left untreated in IL-6-free media (control). Differential gene expression analysis was performed using DESeq2, and we extracted normalized counts and p significance values, adjusted for false discovery rate between controls and IL-33, of a curated list of genes of interest. Genes included host-pathogen interaction (CD14, CD48, NOD2, TLR2, TLR4), cell activation (MHC class II molecules HLA-DRA, HLA-DQA1, HLA-DQA2) and cell adhesion molecules (ICAM1, VCAM1), along with signature genes specific for hMCs (TPSAB1, CPA3, FCER1A, FCER1G, SIGLEC6, SIGLEC8).

**Figure 6:**
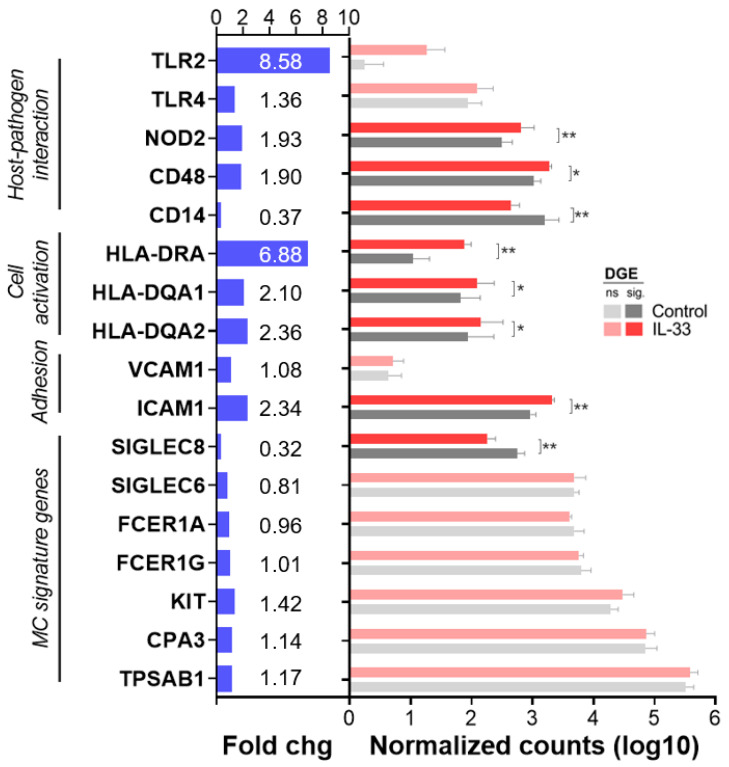
IL-33 promotes the transcription of host-pathogen interaction, cell adhesion and activation genes. hMCs obtained from 4 donors were treated for 24 h with IL-33 (50 ng/mL) or left unstimulated in IL-6-free media (control). After incubation, mRNA was extracted and next-generation Illumina sequencing was performed. Differential gene expression was computed with the R DESeq2 package. Graph shows fold change values from control (left) and normalized counts of selected genes obtained from DESeq2 (median of ratios, right). Statistical analysis was performed with DESeq2′s Wald test for two group comparisons controlled for the false discovery rate (* *p* adj < 0.05, ** *p* adj < 0.001).

As observed in Figure 6, IL-33 treatment enhanced the expression of NOD2 and CD48, non-redundant genes involved in Mtb recognition, and MHC class II molecules, indicators of hMC activation. Furthermore, IL-33-priming significantly increased the expression of adhesion molecules such as ICAM-1(Figure 6).

Thus, cellular activation, as well as increased expression of molecules involved in host-pathogen interaction and cell adhesion, seem to facilitate the hMC-BCG crosstalk.

### 2.7. Human MC Primed with IL-33 Establish Higher Numbers of Rapid Interactions with BCG

**Figure 7:**
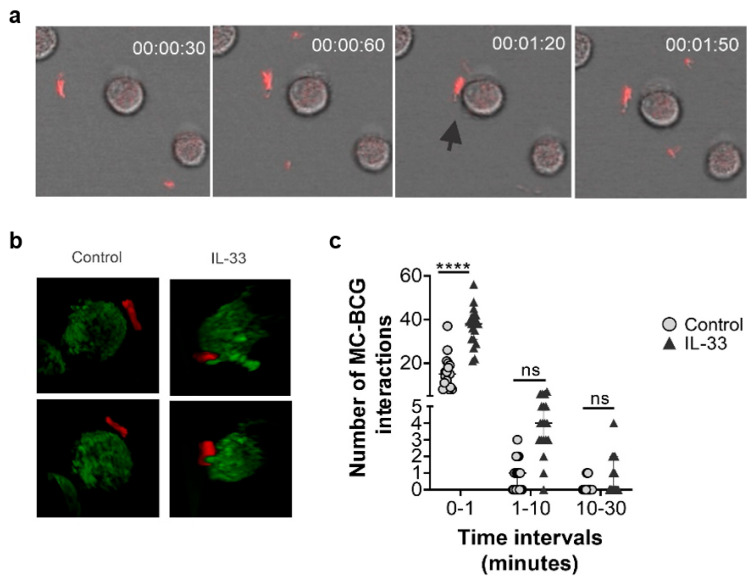
IL-33 treatment increases BCG-hMC interactions. hMCs were treated for 24 h with IL-33 (50 ng/mL) or left unstimulated (control). After incubation cells were washed and left for 30 min in an 8-well chamber at 37 °C. BCG mCherry at a MOI 10:1 was added to wells to perform a live cell imaging for 8 h using confocal microscopy at 37 °C and 5% CO_2_. A 3-dimensional analysis was performed from a representative micrograph of IL-33 pre-treated and untreated cells from obtained videos. (**a**) Live imaging was analysed using FIJI and the number of BCG-hMCs interactions were counted manually in time intervals. A total of 19 cells per power field were counted in each condition (IL-33 treated and untreated). Interactions were considered as single BCG touches at MC membrane or at their protrusions. Arrow shows a representative interaction between BCG and hMCs in control cells. (**b**) Micrographs show in the upper and lower panels different 3D positions from a cell that was left untreated (control) or pre-treated with IL-33 and subsequently exposed to BCG (**c**) Graph is representative of 3 independent experiments. Statistical analysis was performed with one-way ANOVA followed by a Tukey’s multiple comparison test (**** *p* < 0.0001).

Since IL-33-priming increased the expression of the Mtb-binding CD48 receptor, and exerted a broad effect on gene expression of BCG-relevant hMC receptors, we next investigated whether IL-33 pre-treatment could affect the interplay between BCG and hMCs. To this purpose, hMCs were incubated with BCG mCherry (MOI 10:1) at 37 °C with 5% CO_2,_ and were tracked for 8 h with fluorescence 4D confocal microscopy. The number of hMC-BCG contacts at the cell membrane or at cellular protrusions were counted manually from 3 independent experiments (Figure 7a–c, representative videos are included as Supplementary Material Videos S1 and S2). Both untreated cells and IL-33-primed hMCs interacted with BCG at the plasma membrane, with IL-33-primed hMCs showing protrusions surrounding BCG (Figure 7b). However, hMCs primed with IL-33 established significantly higher numbers of rapid (less than 1 min,) interactions with BCG (Figure 7c) compared to untreated hMCs. Although interactions lasting between 1 and 10 min were not significantly different between groups, IL-33 pre-treated cells showed a trend in higher number of contacts compared to untreated controls (Figure 7c).

Thus, IL-33 modulates MC activation and seems to promote early bacteria-MC interactions upon infection.

## 3. Discussion

In the present study, we demonstrated that IL-33 activates hMCs and renders the cells susceptible to BCG stimulation. Furthermore, we showed that IL-33-primed hMCs release inflammatory mediators, display increased CD48 receptor expression, and augment rapid interaction with bacteria upon BCG infection. Thus, our data suggests that an inflammatory microenvironment rich in IL-33 is key to promote a BCG-induced MC response.

MCs are activated upon encountering a variety of virulent pathogens [28]. However, evidence shows that pathogen-associated molecular patters (PAMPs) from virulent bacteria are necessary to activate hMCs [28,29]. For instance, MCs release a wide repertoire of cytokines and chemokines after *Streptocccus equi* exposure, which are not secreted upon incubation to the heat-inactivated bacteria [30]. Similarly, the 19-kDa lipoprotein mediates the release of both pro- and anti-inflammatory cytokines and chemokines [12,31]. HSP70, a known antigen bridging innate and adaptive immunity, is a potent adjuvant inducing the activation of CD8 T cells [32], but also promoting IL-10-mediated anti-inflammatory responses [25]. In our study, HPS70 did not affect hMC activities, possibly because of its lower immunogenicity compared to 19-kDa. The latter is a highly immunogenic cell-wall component of Mtb [33] recognized by TLR2, promoting Mφ apoptosis, secretion of IL-1 β, IL-12, and TNF-α [31], and inducing proliferation of T lymphocytes [34]. Prolonged TLR2 activation (16h exposure) by 19-kDa in Mφ induces an immunosuppressive effect, demonstrated by a decreased response to IFN-γ [35,36]. However, here we showed that hMCs lack or exhibit minimal expression of TLR2, in agreement with published data [37]. Our data demonstrated that hMCs, upon prolonged exposure to 19-kDa (16 h), upregulate CD48, TLR2, but not TLR4, and secrete IL-8, GM-CSF, MCP-1, and IL-13. This is in line with previous findings, showing that Mtb activates rat MCs through CD48 receptor binding [21]. Thus, it seems that hMCs engage virulent antigens, including 19-kDa, via CD48 and TLR2 receptor, similar to Mφ [34].

Since attenuated strains fail to activate hMCs, molecules that potentiate MC activation have attracted interest [17]. These molecules include compound 48/80, IFN-γ and IL-33, which are proposed as vaccine adjuvants [17]. For instance, upon *Staphylococcus aureus* infection, IFN-γ-priming boosts the release of hMC pro-inflammatory mediators including GM-CSF and IL-8 in vitro [27]. Furthermore, compound 48/80 enhances humoral responses, increases secretion of MC-derived TNF-α, and promotes dendritic cell and lymphocyte migration to the lymph nodes in a MC-dependent manner when used as a vaccine adjuvant against *Bacillus anthracis* [38]. Similarly, an in vivo study demonstrated that IL-33, used as an influenza virus vaccine adjuvant, potentiated MC-dependent IFN-γ release and increased survival of mice [39]. In murine Mtb infection, IL-33 increased CD4 T and CD8 T cell recruitment when used in vivo as a vaccine adjuvant [40]. In our study, we showed that upon BCG stimulation, pre-treatment of hMCs with IL-33, but not IFN-γ, enhanced IL-8 and MCP-1 secretion, chemokines involved in neutrophil and monocyte recruitment. This is in accordance with in vitro and in vivo data, showing IL-33 promotion of MC-dependent neutrophil recruitment [41].

Interestingly, IL-33 pre-treatment in hMCs also increased IL-13 secretion upon BCG infection and 19-kDa stimulation. IL-13 contributes to the process of lung fibrosis in human TB [42], since MCs are abundant in fibrotic sites of human pulmonary TB-associated lesions [43]. Therefore, in humans, IL-33 may exacerbate the fibrotic process mediated by IL-13 in a MC-dependent manner.

Although still to be confirmed, the enhanced secretion of hMC mediators could be likely mediated by the IL-33-dependent increased CD48 expression, required for Mtb internalization in MCs [21]. Thus, in a vaccination context, IL-33 may modulate MC-mediated tissue immune responses at the inoculation site, which can trigger TB immunity.

Both IL-33 and IFN-γ treatments did not promote BCG uptake, or support intracellular bacterial growth by hMCs. Although evidence has shown that MCs are capable of phagocytizing bacteria [44,45,46], this lack of internalization may be explained by the BCG deficiency in adhesions required for phagocytosis [1,18,47]. IL-33 is known to potentiate MC adhesion to fibronectin and increase the expression of intercellular adhesion molecule 1 (ICAM-1) and vascular cell adhesion protein 1 (VCAM-1), thus promoting MC adhesion to endothelial cells [48]. In agreement with published data [18,48], we demonstrated in hMCs an increase in ICAM-1 gene expression after IL-33 treatment, together with an upregulation of MHC class II molecules, which might lead to MC-dependent T cell activation and facilitated adaptive immune responses upon BCG stimulation.

In summary, our findings indicate that IL-33 is a potent regulator of MC activities. IL-33 priming of hMCs results in increased hMC-BCG interactions, cell activation and mediator release, thus indicating the key role of IL-33 in regulating the MC-pathogen interplay.

## 4. Materials and Methods

### 4.1. Ethical Statement

Blood samples for cell cultures were obtained from the NHS blood Centre of Manchester under the license REC 2018-2696-5711 approved by the University of Manchester Research Ethical Committee (UREC).

### 4.2. Cellular Culture

hMCs were generated as previously described [29] according to the ethics statement. Briefly, CD117+ haematopoietic progenitors were isolated by immunomagnetic sorting (Miltenyi Biotec, Bergisch Gladbach, Germany) from peripheral blood mononuclear cells (PBMCs). Cells were cultured for 4 weeks in Iscove Modified Dulbecco medium (IMDM) with GlutaMAX-I supplemented with 50 µmol/L β2-mercaptoethanol, 0.5% BSA, 1% Insulin-Transferrin-Selenium, 100 U/mL penicillin, 100 µg/mL streptomycin (Invitrogen, Carlsbad, CA, USA), human IL-6 (50 ng/mL; PeproTech, Rocky Hill, NJ, USA), human IL-3 (10 ng/mL; PeproTech), human stem cell factor (SCF, 100 ng/mL; PeproTech), and in the above medium without IL-3 from week 4 thereafter. hMCs, cells were tested for purity and maturity after 8 weeks culture (Supplementary Figure S1). THP-1 monocyte cellular line (ATCC) was cultured in RPMI (Sigma-Aldrich, St. Louis, MO, USA) medium containing L-glutamine supplemented with 10% foetal bovine serum (FBS; Invitrogen, Waltham, MA, USA) at 37 °C and differentiated into macrophages at a concentration of 5 × 10^5^ cells/mL in a flat bottom plate using phorbol 12-myristate 13-acetate (PMA) (100 µM; Thermo Fisher Scientific, Waltham, MA, USA) for 2 h. Cells were then washed and rested in RPMI for at least 1 h before being used.

### 4.3. Bacterial Cultures

BCG-LuxG13, BCG-mCherry (gifts from Professor Brian Robertson, Imperial College London, UK) and BCG (Danish 1331 substrain) were cultured in 7H9 broth (7H9, BD Biosciences, Franklin Lakes, NJ, USA) supplemented with 0.05% Tween 80, 0.2% glycerol and 10% oleic albumin dextrose catalase (OADC; BD Biosciences, San Jose, CA, United States) for 2 weeks until reaching an optical density (O.D) of 0.5. BCG-LuxG13 was produced by transformation of Danish strain with a bacterial luciferase encoding vector (pMV306hsp + LuxAB + G13 + CDE, KanR) as described previously. Hygromycin (100 ug/mL; Sigma-Aldrich) was added to BCG-mCherry cultures and Kanamycin (50 µg/mL; Sigma-Aldrich) was added to BCG-Lux cultures. Bacterial suspensions were washed using PBS-tween (0.05%; Sigma-Aldrich) and diluted in glycerol (15%; Sigma-Aldrich) to prepare homogenous concentrated stocks. Bacterial stocks were frozen down with liquid nitrogen and kept at −80 °C. After at least 24 h, 3 stocks were randomly selected, thawed, and diluted in serial concentrations before being cultured in 7H11 agar plates (7H11; BD Biosciences) for colony-forming units (CFU) and incubated for 15 days at 37 °C. Colony counting was recorded after 7–15 days and final count was taken at day 15. Corresponding calculations were done to obtain bacterial concentrations using the mean of 3 representative stocks. Before infections, a single stock was thawed at room temperature and diluted accordingly.

### 4.4. Cellular Priming, Stimulation and Infection

hMCs at a density of 5 × 10^5^ cells/mL were incubated with recombinant IL-33 (50 ng/mL) (PeproTech) or IFN-γ (50 ng/mL) (PeproTech) for 24 h at 37 °C and 5% CO_2_, and washed 3 times before further stimulation. For BCG infection, naïve or cytokine-treated hMCs or THP-1-derived macrophages were washed 3 times to remove antibiotics and plated at a density of 5 × 10^5^ cells/mL in supplemented IMDM without antibiotics. Bacteria were diluted from stocks at a final MOI of 10:1 in the same media without antibiotics. Bacterial suspension was added to plates containing cells and incubated at 37 °C, 5% CO_2_ for 1 h for degranulation, and 16h for cytokine secretion. For Mtb-antigen stimulation, hMCs at a density of 5 × 10^5^ cells/mL were incubated with 19-kDa lipoprotein (10 µg/mL) (Lionex GmbH, Braunschweig, Germany) and with HSP70 (10 µg/mL) (Lionex GmbH) for 16h to evaluate cytokine release. hMCs (5 × 10^5^ cells/mL) and 19-kDa (10 µg/mL) (Lionex GmbH) were incubated for 1 h to investigate degranulation and receptor expression.

### 4.5. Flow Cytometry

hMCs were washed and stained with anti-human CD63 (clone H5C6; BioLegend, San Diego, CA, USA), CD107a (clone H4A3; BioLegend), CD48 (, clone BJ40; BioLegend), TLR2 (AL547, clone QA16A01; BioLegend), and TLR4 (clone HTA125; BioLegend) in FACS buffer for 30 min at 4 °C. Fluorescence minus one (FMO) was used as a staining control, compensation was performed using compensation beads (OneComp eBeads; Thermo Fisher Scientific). Cells and controls were acquired using a BD LSR-II flow cytometer (BD Biosciences). Data was analysed with FlowJo software (version 10.4.2, BD Life Sciences, Ashland, OR, USA) and expressed as percentage of positive cells.

### 4.6. Cytokine and Chemokine Secretion

Cellular supernatants were collected after 16h and used to determine IL-8, GM-CSF, MIP-1α, MCP-1, TNFα, IL-10, IL-13, and IL-1β concentrations. Cytokine and chemokine concentrations were measured using a multiplex capture bead array assay (CBA; BD Biosciences) by flow cytometry (FACSVerse; BD Biosciences) following manufacturer’s instructions. Data were analysed with FCAP Array v3.0 software (BD Biosciences).

### 4.7. β-Hexosaminidase Assay

β-hexosaminidase release was measured as previously reported [29]. Briefly, hMCs (0.5 × 10^6^ cells/mL) were stimulated with BCG, or pre-sensitized with anti-IgE and stimulated with anti-IgE antibodies for 1 h. Supernatants were harvested, and cells were treated with Triton X-100 (1%; Sigma-Aldrich). Both cell lysates and supernatants were incubated with p-nitrophenyl N-acetyl-beta-D-glucosamine (1 mmol; Sigma-Aldrich) in citrate buffer (0.05 M; pH 4.5) for 2 h at 37 °C. After incubation, samples were treated with sodium carbonate (0.05 M, pH 10.0) and O.D was taken at 405 nm using spectrophotometric analysis. Β-hexosaminidase was expressed as percentage of total release.

### 4.8. BCG Intracellular Growth and Internalization

To evaluate bacterial growth, hMCs or THP-1 cells (both at 0.5 × 10^6^ cells/mL) were washed to remove antibiotics and incubated with BCG-Lux using a MOI of 10 for 4 h in supplemented media without antibiotics. Cells were then washed to remove extracellular bacteria, and incubated for up to 6 days (150 h). Luminescence was measured at different time points as relative light units (RLUs) with a microplate Luminometer (LUMIstar Omega; BMG Labtech, Ortenberg, Germany). Increase of RLUs was considered as increase of bacterial growth. To evaluate bacterial uptake, hMCs or differentiated THP-1 macrophages (2 × 10^4^ cells) were placed in 8-well IbidiTreat chambers (Ibidi GmbH, Gräfelfing, Germany) at 37 °C and 5% CO_2_ prior to infection. BCG mCherry was added at a MOI 10:1 for 2 h and hMCs were fixed with paraformaldehyde 4%. Cellular membranes were stained with wheat germ agglutinin (WGA), conjugated to Alexa Fluor 488 (Invitrogen). Confocal images were collected using a Leica TCS SP8x inverted confocal microscope (Leica Microsystems GmbH, Wetzlar, Germany). BCG mCherry was excited at 590 nm in a WLL laser (80%) and detected at 598 to 690 nm on a HyD point detector. WGA-Alexa Fluor 488 fluorescence was excited with the 488 nm Argon laser (19%) and collected on a HyD detector at 492 to 550 nm. Images were analysed using Fiji [49].

### 4.9. Confocal Microscopy

Naïve or cytokine-treated hMCs (2 × 10^4^ cells) were placed in 8-well IbidiTreat chambers (Ibidi GmbH) in Hank’s buffer supplemented with IL-6 (50 ng/mL) and SCF (100 ng/mL) for 30 min prior to infection at 37 °C and 5% CO_2_. BCG mCherry was added at a MOI 10:1 and chambers were taken to confocal microscope pre-set at 37 °C and 5% CO_2_. Live cell imaging was performed using a Leica TCS SP8x inverted confocal microscope, equipped with a tuneable white light laser (WLL), a 40×/0.85 dry objective, and HyD hybrid detectors. BCG mCherry was excited at 590 nm with WLL laser (80%) and detected at 598 to 690 nm on a HyD point detector. Confocal 3D stacks were acquired with a z-depth of 8 µm using Leica LASX software and stacks were taken at 30 s interval. Image stacks were sum projected into a single plane image and analysed using Fiji [49]. BCG-hMC contact events were counted manually with a total of 19 cells per field per experiment.

### 4.10. mRNA Extraction, Sequencing and Differential Gene Expression Analysis

hMCs (0.5 × 10^6^ cells/mL) from four separate donors were left overnight at 37 °C in IMDM with GlutaMAX-I culture media, supplemented with 50 µmol/L β2-mercaptoethanol, 0.5% BSA, 1% Insulin-Transferrin-Selenium, 100 U/mL penicillin, 100 µg/mL streptomycin and 100 ng/mL human SCF (IL-6-free media). Subsequently, cells were either incubated for 24 h at 37 °C and 5% CO_2_ in IL-6-free media, or in IL-6-free media with added 50 ng/mL human IL-33 (PeproTech). After incubation, mRNA was extracted with a commercially available kit (RNAeasy micro kit; Qiagen, Hilden, Germany) according to the manufacturer’s protocol. Next generation sequencing on isolated mRNA was conducted by the University of Manchester Genomic Technologies Core Facility using the Illumina HiSeq^®^4000 system (Illumina Inc., San Diego, CA, USA). Quality control and data pre-processing was performed by the University of Manchester Bioinformatics Core Facility. Reads were mapped to the human genome using the GRCh38/hg38 reference and Gencode v.36 was used for annotation [50]. Differential gene expression analysis from raw counts was computed with the DESeq2 v1.30.1 package in R [51].

## 5. Conclusions

Although in vitro hMCs are not susceptible to a BCG-induced activation, a microenvironment rich in IL-33 increases MC responsiveness to BCG, likely by upregulating the expression of the CD48 receptor. Thus, IL-33 could be used to enhance the immunogenicity of the BCG vaccine, and to improve the BCG therapeutic activity in non-TB pathologies.

## Supplementary Materials

The following supporting information can be downloaded at: https://www.mdpi.com/article/10.3390/ijms23147549/s1.
