# Supplementary material for: Bacillus Calmette–Guérin-Induced Human Mast Cell Activation Relies on IL-33 Priming
Source: Int J Mol Sci. 2022 Jul 7;23(14):7549. doi: 10.3390/ijms23147549 (PMC9320129; doi:10.3390/ijms23147549)
Supplement: Supplementary file 1 [file ijms-23-07549-s001.zip › ijms-1766473-supplementary.pdf]

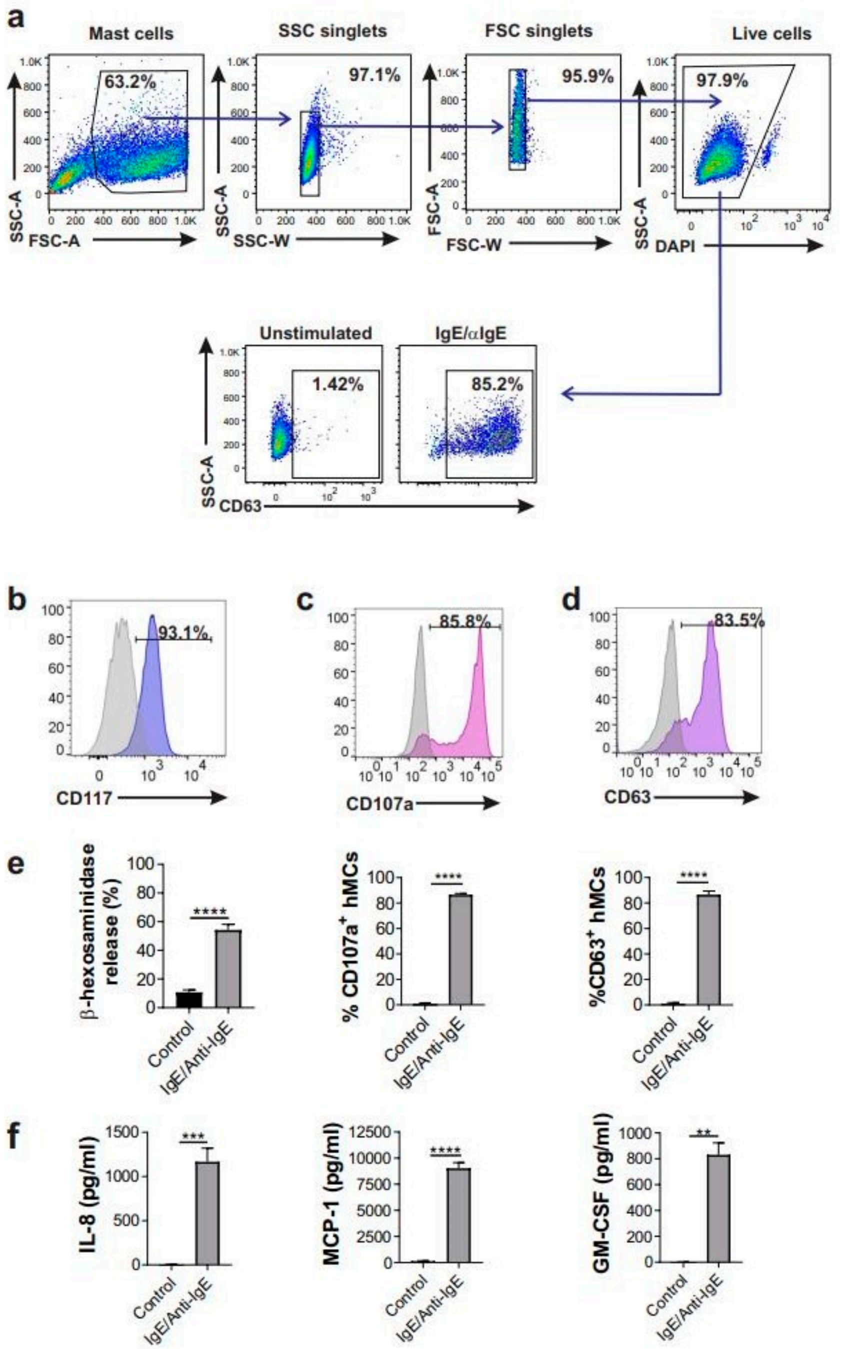

Supplementary Figure S1. Blood progenitors-derived human mast cells: Gating strategy, purity and maturity. Hematopoietic hMC progenitors were isolated from PBMCs from different donors and differentiated in the presence of cytokines into mature hMCs. After 8 weeks of differentiation and maturation, hMCs were analyzed for purity and maturity before experiments. hMCs were sensitised overnight with IgE (1 µg/mL), washed and either left untreated (control) or stimulated with anti-IgE antibody (1 µg/mL) for 1h to investigate degranulation, or 8h to analyze cytokine secretion. After 1h IgE incubation cells were stained with anti-CD117, anti-CD63 and anti-CD107a antibody and analysed by flow cytometry**. (a)** Gating strategy used for flow cytometry analysis. Cells were gated using side scatter (SSC-A) and forward scatter (FSC-A) parameters. Single cells were selected using SSC- A/SSC-W and FSC-A/FSC-W parameters, while dead cells were discriminated using DAPI. Live cells were used for further analysis including degranulation ($CD63^+$ cells) and cell surface markers. Lower panel shows a representative example of cellular gating on CD63 positive cells compared to an unstimulated control. **(b, c and d)** Histograms show CD117 (blue), CD107a (pink) and CD63 (purple) positive cell counts compared to unstained controls (grey). Graphs show hMC degranulation as percentage of **(c)** $CD107a^+$ and **(d)** $CD63^+$ hMCs. **(e)** Degranulation was confirmed with β- hexosaminidase release assay. **(f)** After 8h IgE incubation, supernatants were collected and IL-8, MCP-1 and GM-CSF release was measured. Each graph shows a representative experiment from 3 independent experiments. 3 different donors were used for hMC experiments from 7 different cell cultures. Statistical analysis was done using *t*-test (*p*= **<0.01, ***<0.0001, , ****<0.00001).

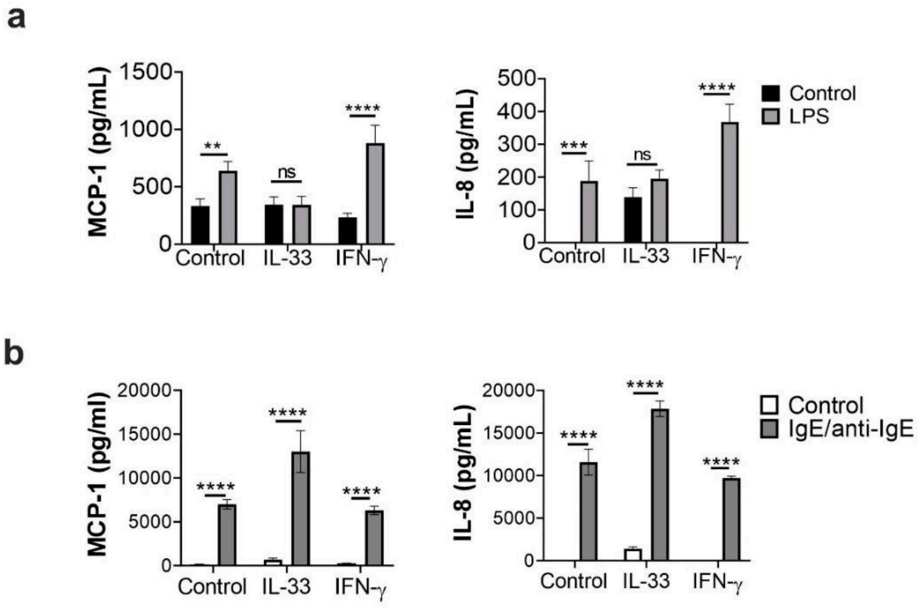

Supplementary Figure S2. hMC mediator secretion upon LPS stimulation and IgE-FcεRI crosslinking is enhanced by IL-33. hMCs were treated with IL-33 (50 ng/ml) or IFN-g (50 ng/ml) for 24h, or left untreated (control). **(a)** Cells were washed and stimulated with LPS for 16 h or **(b)** sensitized overnight with IgE (100 µg/ml), and stimulated with an anti-IgE (100 µg/ml) antibody for 16h. After incubation, supernatants were collected and MCP-1 and IL-8 were measured using the CBA multiplex assay. Each graph shows a representative experiment from 3 independent experiments. Statistical analyses were performed using one-way ANOVA analysis followed by a Tukey's multiple comparison test (****p<0.0001, ***p<0.001, **p<0.01).
